# Supplementary material for: A Systematic Review on Processed/Ultra-Processed Foods and Arterial Hypertension in Adults and Older People
Source: Nutrients. 2022 Mar 13;14(6):1215. doi: 10.3390/nu14061215 (PMC8955286; doi:10.3390/nu14061215)
Supplement: Supplementary file 1 [file nutrients-14-01215-s001.zip › nutrients-1583961-supplementary.pdf]

Supplementary Materials

Table S1. PICOS acronym used in the design of the study (n = 9).

Acronym	Description
P (Population)	Adults (20 to 59 years of age) and/or older people (60 years of age or older)
I (Intervention)	High consumption of processed and ultra-processed foods based on the NOVA classification
C (Control)	Low consumption of processed and ultra-processed foods based on the NOVA classification
O (Outcome)	Arterial hypertension defined based on any diagnostic criteria
S (Study design)	Observational (cohort, case-control and cross-sectional) and intervention studies

Table S2. Methodological quality of studies selected for present review (n = 9).

**Modified Newcastle Ottawa**	**Selection**				**Comparability**	**Outcome**				**Classification**
item	1	2	3	4	1	1	2		Total	
Conceição et al. (2018) [19]	☆	☆	-	☆☆	-	-	☆		☆☆☆☆☆	Good
Smiljanec et al. (2020) [21]	-	-	-	☆☆	☆	-	☆		☆☆☆☆	Fair
Martinez-Peres et al. (2021) [24]	☆	☆	☆	☆☆	☆	-	☆		☆☆☆☆☆☆☆	Good
Nardocci et al. (2020) [3]	☆	☆	☆	☆☆	☆	-	☆		☆☆☆☆☆☆☆	Good
Martínez-Steele et al (2019) [22]	☆	☆	☆	☆☆	☆	-	☆		☆☆☆☆☆☆☆	Good
**Newcastle-Ottawa**	**Selection**				**Comparability**	**Outcome**				**Classification**
item	1	2	3	4	1	1	2	3	Total	
Rezende-Alves et al. (2020) [2]	☆	☆	☆	☆	☆	-	☆	☆	☆☆☆☆☆☆☆	Good
Monge et al. (2021) [23]	-	☆	☆	☆	☆	-	☆	☆	☆☆☆☆☆☆	Good
Mendonça et al. (2017) [1]	☆	☆	☆	☆	☆	-	☆	☆	☆☆☆☆☆☆☆	Good
Scaranni et al. (2021) [20]	☆	☆	☆	☆	☆	-	☆	☆	☆☆☆☆☆☆☆	Good
